# Risk factors for bleeding complications after percutaneous dilatational tracheostomy: a 10-year institutional analysis

**DOI:** 10.1186/cc14293

**Published:** 2015-03-16

**Authors:** K Pilarczyk, G Marggraf, M Dudasova, E Demircioglu, D Wendt, B Huschens, H Jakob, F Dusse

**Affiliations:** 1 West German Heart Center Essen, University Hospital Essen, Germany; 2 University Hospital Essen, Germany

## Introduction

Percutaneous dilatational tracheotomy (PDT) is the standard airway access in critically ill patients who require prolonged mechanical ventilation. Bleeding complications after PDT are infrequently observed but have a tremendous impact on further clinical course CFA risk stratification for patients scheduled for PDT.

## Methods

We retrospectively reviewed the records of all patients who underwent PDT (using the Ciaglia technique with bronchoscopic guidance) on our cardiothoracic ICU between 2003 and 2013. Patients were stratified into two groups: patients suffering from acute moderate, severe or major bleeding (Group A) and patients who presented none or only mild bleeding (Group B).

## Results

A total of 1,001 patients (46% male, mean age 68.1 years) that underwent PDT were analyzed. In the majority of patients, no or only mild bleeding during PDT occurred (none: 425 (42.5%), mild: 488 (48.8%)). In 84 patients (8.4%), bleeding was classified as moderate. Three patients suffered from severe bleeding, only one major bleeding with need for emergency surgery was observed. Study groups showed significant differences in Simplified Acute Physiology Score (SAPS) on the day of PDT (Group A: 47.0 ± 13.1, Group B: 32.9 ± 11.2, *P *= 0.042), renal replacement therapy on the day of PDT (Group A: 53 (60.2%), Group B: 439 (48.1%), *P *= 0.026), presence of coagulopathy (Group A: 48 (54.5%), Group B: 393 (43.0%), *P *= 0.043), platelet count (Group A: 91.6 ± 59.2, Group B: 111.5 ± 79.8 × 1,000/μl, *P *= 0.037), fibrinogen levels (Group A: 373.6 ± 159.1, Group B: 450.6 ± 259.0 mg/dl, *P *= 0.012), proportion of PDTs performed by residents (Group A: 72 (81.8%), Group B: 632 (69.2%), *P *= 0.034) and moderately to very difficult PDT (Group A: 31 (35.2%), Group B: 141 (15.4%), *P *= 0.001). Using logistic regression analyses, difficult PDT, low-experienced operator, SAPS >40 and low fibrinogen were independent predictors of relevant bleedings after PDT.

## Conclusion

Periprocedural bleeding complications during PDT are rare. However, low fibrinogen levels as well as difficult PDT, low-experienced operator and SAPS >40 are associated with an increased risk for bleeding complications. Therefore, preprocedural risk evaluation for bleeding complications should include these factors and further studies are necessary to prove whether modification of risk factors - for example, substitution of fibrinogen prior to PDT - is able to reduce incidence of bleeding complications.

